# A multi-layer encoder prediction model for individual sample specific gene combination effect (MLEC-iGeneCombo)

**DOI:** 10.1371/journal.pcbi.1013547

**Published:** 2025-10-03

**Authors:** Yun Shen, Kunjie Fan, Birkan Gökbağ, Nuo Sun, Chen Yang, Lijun Cheng, Lang Li

**Affiliations:** 1 Department of Biomedical Informatics, College of Medicine, The Ohio State University, Columbus, Ohio, United States of America; 2 Department of Physiology and Cell Biology, College of Medicine, The Ohio State University, Columbus, Ohio, United States of America; University of Wisconsin Madison, UNITED STATES OF AMERICA

## Abstract

Using data from gene combination double knockout (CDKO) experiments, top ranked synthetic lethal (SL) gene pairs were highly inconsistent among different SL scores. This leads to a significant concern that SL prediction models highly depend on SL scores. In this paper, we introduce a new gene combination effect (GCE) measurement, log-fold change of dual-gRNA expression before and after CRISPR-cas9 lentivirus transfection. We show it is a direct and highly consistent measurement of GCE in all CDKO experiments. We therefore develop a multi-layer encoder model for individual sample specific GCE prediction, MLEC-iGeneCombo. Under a deep learning framework, MLEC-iGeneCombo is a systems biology model that contains sample specific multi-omics encoder, network encoder and cell-line encoder. For the first time, MLEC-iGeneCombo predicts GCE for a new cell. Using data from 18 CDKO experiments, MLEC-iGeneCombo achieves an average GCE prediction performance, 71.9%. All three encoders significantly improve the model’s prediction performance (p≤0.01), and their combined use yields the best GCE prediction performance. Our source code is available at https://github.com/karenyun/MLEC-iGeneCombo.

## Author summary

Understanding how pairs of genes interact is critical for deciphering the complexities of genetic diseases and cancer therapies. Our work focuses on accurately measuring how the combination of two genes affects cell survival, especially when both genes are disrupted. Traditionally, scientists have studied this phenomenon—called synthetic lethality—by evaluating indirect measures, leading to inconsistent and conflicting results. To address these inconsistencies, we adopted a direct and reliable measure in a gene combination double knockout experiment: log fold change of sgRNA expression. In our systematic review of existing experimental methods, we found that log fold change was the most consistent and comparable measure across various studies. Building upon this insight, we developed a new model capable of predicting gene combination effect in a new cell. Hence, its gene combination prediction is cell specific. Our work lays the foundation for personalized combinatory target discovery in cancer and other complex diseases.

## Introduction

Gene combination effect refers to the result of genetic effect between two genes within a cell. Initially, the concept of gene combination effect was observed in model organisms, including fruit flies, in which researchers noted mutations in the bar or glass genes occurring individually but never together [1–2]. Other gene combination effect examples are paralog genes from the same family frequently share function, such as CCNL1/CCNL2 and CDK4/CDK6 in human lung cells [3]. Gene combination effect is also characterized by the redundancy of signaling pathways. The relationship between BRAC and PARP is a classic example. In humans, these two genes control two different paths in the DNA damage pathway [4]. Clustered regularly interspaced short palindromic repeats (CRISPR)-Cas9 gene combination double knockout (CDKO) is a cutting-edge high throughput technology capable of investigating the effects of combinations of thousands of gene pairs among hundreds of genes. Biologically, synthetic lethality, one type of gene combination effects, has received a great deal of attention in CDKO experiments exploring the effect of gene combinations. Conceptually, in an SL gene pair, the cell dies when the function of both genes is disrupted but remains viable when the function of only one of the genes is compromised. Computationally, SL is defined as the larger effect of gene combination than the additive effect from two genes.

CRISPR-cas9 CDKO experiments typically were designed to include three to four guided RNAs, i.e. gRNAs, for knocking out a gene. The effect of gene knockout, that is, the effect of a single gene, on cell viability is estimated by the average log fold change (LFC) of its gRNAs from baseline (T0) to the end of the screening experiment (Tend). The smaller the LFC, the stronger the effect of gene knockout on cell viability. Defined similarly to the single gene effect, the effect of gene combination knockout, i.e., the effect of gene combination, is estimated by the median of the LFC from the dual-gRNAs from two genes.

We carefully reviewed CDKO experiments and the concepts of single gene and gene combination effects [5] and SL effects. We found that enormous difference on how SL were defined and calculated among these published CDKO experiments. In S1 Table, the difference in SL calculation was highlighted in five components: dual-gRNA count filtering, dual-gRNA count normalization, dual-gRNA effect, dual-gRNA effect normalization, and SL score calculation. For the sake of simplicity, we assume a CDKO experiment has dual-gRNAs measured at day 0 (i.e., beginning) and day T (i.e., end). In dual-gRNA design, it includes dual-gRNAs targeting on two genes, dual-gRNAs targeting on no-gene (i.e., control dual-gRNAs), and dual-gRNAs targeting on one gene and one control gRNAs.

- Dual-gRNA count filtering is the 1st step in sequencing data processing. Its goal is to remove lowly expressed gRNA either at day 0 or day T. Among these CDKO experiments, some removed dual-gRNAs with lower counts, but with different thresholds, e.g., 100, 50, 10, or 32. Some filtered out dual-gRNAs with 0 count, and some did not speculate the detail.
- Dual-gRNA count normalization is to make sure the samples are comparable under different treatment conditions or replicates within a condition. We have seen three different methods, normalization to the total dual-gRNA reads, normalization to the total cell populations, and median ratio normalization.
- Dual-gRNA effect is the most consistent measure, and it is all calculated in LFC of dual-gRNA counts between day 0 and day T among all CDKO experiment.
- Dual-gRNA effect normalization removes the control dual-gRNA effect from targeted dual-gRNA effect. Apparently three out of ten CDKO experiments did not consider the control dual-gRNA effect. Their counter argument is that it is difficult to define and identify the true control dual-gRNAs.
- SL calculation methods show the most diversity. Firstly, there was a fundamental difference in how SL was defined. Some SLs were defined as the difference between dual-gRNAs effect for two genes and dual-gRNAs effect for one gene or no gene [3,6–8]. Some SLs were calculated as the difference between dual-gRNAs effect for two genes and additive effect from two dual-gRNA effects on targeting on each individual gene [9–13]. In [14], more diverse SLs were defined and calculated. Secondly, the statistical methods in inferring SL are even more diverse than SL definition itself, including frequentist methods such as, t-test, Mann Whitney U-test, and linear regression; posterior probability from Bayes regression; and false discovery rate under empirical Bayes. Only one method, GEMINI [15], presented a comprehensive hierarchical Bayes model framework in SL estimation and inference. All the other methods were heuristic statistical methods. Technically, none of these ten CDKO experiments implemented the exact same statistical methods.

In the recently publication [16] compared these five representative approaches in SL scoring, finding that the top 10% of gene pairs for each algorithm had only 1.21% overlap across all five approaches.

These evidence in S1 Table strongly suggest that SL practically is enormously inconsistent and should not be an ideal measurement for gene combination effect. On the other hand, S1 Table also demonstrates a striking consistency among all CDKO experiment, dual-gRNA effect was all measured in LFC of dual-gRNA counts between day 0 and day T. We therefore decide to use LFC as the primary measure of gene combination effect. Biologically, SL characterizes whether the effect on cell viability of two genes in combination is greater than their additive effects, or sometimes individual gene effect. SL is an indirect measurement of two genes’ effect on cell viability. Unlike SL, gene combination effect by LFC of dual-gRNA is a direct measurement of two genes’ effect on cell viability.

Although CRISPR-Cas9 CDKO can investigate thousands of gene pairs among hundreds of genes, it cannot reach and screen tens of millions of gene pairs across the genome [13]. This is the primary motivation that machine learning (ML) and deep learning (DL) models were developed for genome wide SL prediction. All the current ML and DL models were trained from SL data collected from the SynlethDB [17]. However, this synthetic lethality database [18] incorporated only half of the SL data of published CDKO experiment data [13] and collected only a fraction of SL and non-SL gene pairs among the CDKO experiments it did include. Noticeably, SynlethDB failed to recognize that different CDKO experiments employed different SL calculation approaches. Its SL scores in the database were not consistently defined and were not directly comparable. In contrast, SLKB [16] curated all 12 CDKO experiments, including gene combination effect data on 280,483 gene pairs from 22 cell lines. This is an ideal data set in developing predictive models for gene combination effect.

Using SynLethDB data, current SL predictive models were based on population features rather than cell-specific context. Thus, its SL interpretation remains unclear. For instance, the SL of a gene pair in at least one cell may not indicate it to be SL in all cells. Table 1 summarizes these population-based model. They typically have two feature types: pathway/network features, including protein-protein interaction (PPI) networks, gene ontology, and signaling pathway graph, and omics features, including gene expression (GE), copy number variation (CNV), essentiality (ES) from CRISPR or small interfering RNA (siRNA) screening, and mutations (MU) from genomics data. Some deep learning methods, especially graph neural network (GNN) methods, have been widely utilized to improve SL prediction. GNN approaches excel at integrating input graph topology and node attributes to learn informative representations for downstream predictions [19].The first proposed GNN-based model for SL prediction, DDGCN [20], considered only the SL network as the input feature. GCATSL [21], a more advanced type of GNN employed a graph attention network (GAT) to integrate multiple biological sources, including gene ontology (GO) and protein-protein interaction (PPI) as node attributes for improved performance. Both KG4SL [22] and PiLSL [23] considered knowledge graphs to facilitate interpretations, and PiLSL included omics features. By constructing enclosing subgraphs for each pair of genes and utilizing attentive embedding propagation, PiLSL has achieved state-of-the-art performance for the population based SL prediction.

**Table 1:** Machine- and deep-learning models for the prediction of synthetic lethality (SL).

Method	Network features			Omics features			
	PPI	GO	KG	GE	CNV	ES	MU
Mashup	Y	Y					
Coupled matrix factorization (CMF)				Y	Y	Y	Y
Matrix factorization to synthetic lethality (SL*MF)				Y	Y	Y	Y
Dual-dropout graph convolution network (DDGCN)	Y			Y	Y	Y	Y
Graph context attention network for synthetic lethality (GCATSL)	Y	Y					
Knowledge graph for synthetic lethality (KG4SL)	Y	Y	Y				
Pairwise-interaction learning-based graph neural network for synthetic lethality (PiLSL)			Y	Y	Y	Y	Y
MVGCN-iSL	Y			Y	Y		Y
MLEC-iSL	Y	Y		Y	Y		Y

CNV (copy number variation), ES (essentiality, or CRISPR or siRNA screening), GE (gene expression), GO (gene ontology), KG (knowledge graph of signaling pathways), MU (genetic mutations), PPI (protein-protein interaction)

Two recent published SL predictive models, multi-view graph convolutional network for individual sample specific SL prediction (MVGCN-iSL [24]) and multi-layer encoder for individual sample specific SL prediction (MLEC-iSL [25]) included sample specific omics and network features and performed sample specific SL prediction. Although MVGCN-iSL and MLEC-iSL were developed using the consistent SL calculation method and data from [4], these two models are not scalable to predict SL genes for a new cell.

In this paper, in order to avoid inconsistency among SL scores generated by variable SL calculation methods, we choose LFC of the dual-gRNA as the primary measure of gene combination effect. LFC has been consistently used and reported among all CDKO experiments. Unlike SL, gene combination effect measured by LFC is a direct robust measurement of two genes’ effect on cell viability. For the first time, we develop a multi-layer encoder model that can predict individual sample specific gene combination effect in LFC, i.e., MLEC-iGeneCombo. Unlike existing population-based predictive models or individual sample specific prediction models, MLEC-iGeneCombo predicts gene combination effect for a new cell.

## Materials and methods

### Dataset

#### Gene combination double knockout (CDKO) experiment data.

We adopted a subset of the dataset obtained from gene combination double knockout (CDKO) experiments [16] in the recent developed synthetic lethality knowledge base (SLKB), which includes 11 CDKO experiments, 22 cell lines, and LFC from 280,488 non-SL gene pairs. Our subset includes CDKO experimental data for 189,265 gene pairs in 17 cells and an extra cell-line, SAOS-2 cell, which contains 1,540 gene pairs. Additionally, we conducted subset testing using only six cell lines, as these contained non-overlapping gene pairs or genes relative to the training set. The S2 Table shows the detail information of 18 cell-lines.

#### Gene combination effect (GCE) definition and CDKO experiment data processing.

In each CDKO experiment, cells were infected with a lentivirus that contained a library of dual gRNAs at Day 0, at which time the distribution of gRNA sequences was approximately even. After a period of usually three to four cell cycles, at Day T_end_, significant suppression of cell viability by the dual-gRNA-guided CRISPR-cas9 gene knockout was reflected in a decrease in the corresponding dual-gRNA sequences from Day T_0_. Thus, the fold change (FC) in the dual-gRNA sequences from Day T_0_ to Day T_end_ indicated the effect on cell viability of the dual gRNAs for a gene combination.

The count of each gRNA pair was first normalized to the sum of the total counts across all time points; a pseudo counts of 32 was applied when the dual gRNA data below this threshold; and the LFC for each gRNA was calculated between the final and initial time point counts for each replicate. The GCE was calculated as the median of the LFCs of all corresponding dual-gRNAs. The S1 Appendix detail setup of CDKO experiments, processing of gRNA data, and calculation of gene combination effect.

#### Multi-omics data.

We acquired gene expression (GE) data copy number variation (CNV) data from the Cancer Cell Line Encyclopedia (CCLE) database [26] and CRISPR essentiality (ES) data from the Cancer Dependency Map portal (DeepMap) [27]. In our approach, multi-omics data include GE, CNV, and GE data across all cell lines. Both population and cell-specific multi-omics data are used in our model. Genome wide multi-omics data are processed in principal component analysis (PCA) to represent the cell lines effectively.

#### Biological network data.

We incorporated physical protein-protein interactions from the BioGRID dataset [28] and excluded gene nodes not expressed in the selected cell lines. Specifically, we applied a filtering step to retain only **physical interactions (experimental system type = “physical”)** from BioGRID. As input for the gene nodes, we applied population-based multi-omics data across all cell lines using PCA.

### Proposed method

#### Overview.

**Fig 1:**
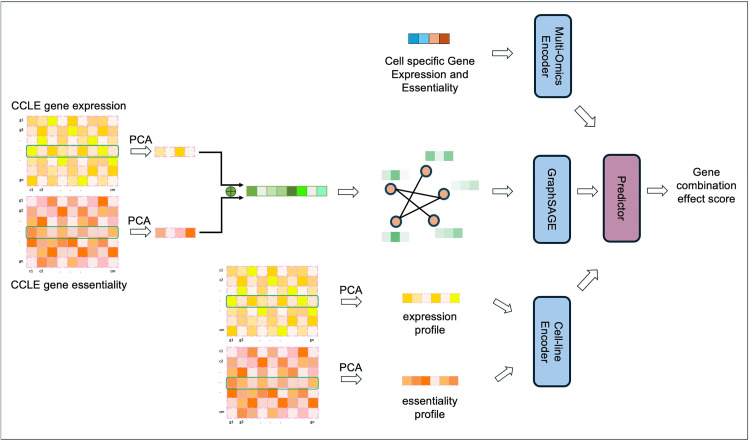
Overview of the proposed multi-layer encoder model, MLEC-iGeneCombo, for predicting gene combination effects. The model comprises three components: a multi-omics encoder, a network encoder, and a cell-line encoder. The multi-omics encoder uses gene expression and essentiality data from the specific cell line. The network encoder incorporates gene population features into the PPI network as node features. The cell-line encoder processes population-level features of the cell lines as input.

We proposed a new multi-layer encoder comprising multi-omics, network, and cell-line encoders for our MLEC-iGeneCombo model, designed to predict GCEs. The framework is illustrated in Fig 1. Our approach takes cell-specific multi-omics data as input to the multi-omics encoder, gene profiles as nodes in the graph neural network, and cell line profiles as input to the cell-line encoder. The final gene combination effect score is predicted based on the combined output features from these three encoders.

#### Multi-omics encoder.

**Fig 2:**
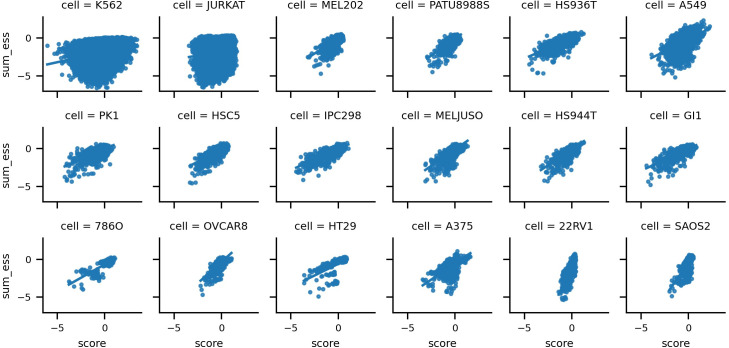
Correlation between gene essentiality and gene combination score.

**Fig 3:**
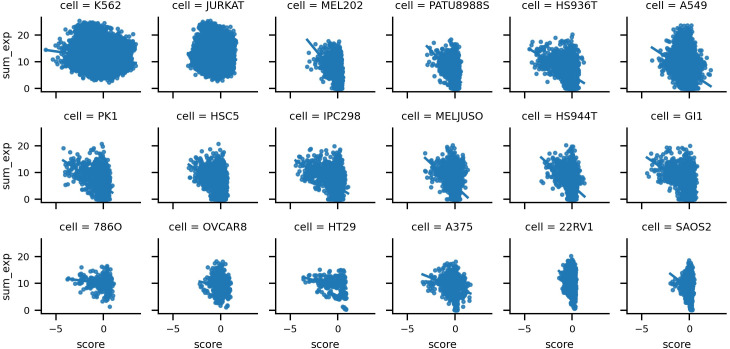
Correlation between gene expression and gene combination score.


We leveraged two distinct types of omics data: gene expression and gene essentiality, and observed their robust positive correlation across most cell lines shows in Fig 2. Particularly noteworthy, 22Rv1 exhibited pronounced linear correlation, whereas JURKAT and K562, characterized by a wider array of samples, showed no discernible positive linear correlation. In contrast, gene expression values demonstrated modest negative correlation shows in Fig 3. Consequently, we incorporated both these omics values as inputs, anticipating their collective efficacy in accurately predicting the final score. To extract gene omics features, we used a multi-layer perceptron (MLP) with 32 hidden layers to encode the multi-omics value for the gene pair:

$$F_{omics}=MLP([X_{exp}⊕X_{ess}⊕Y_{exp}⊕Y_{ess}])$$ (1)

where the MLP is a fully connected feedforward neural network comprising multiple layers of perceptrons. Each layer is defined as σ(WX+b), where X is the input, W and b are the learnable weights, and σ is the activation function. For example, LeakyRelu(·)=max(·, 0)+negative_slop·min(0, x). $X_{exp}$ and $Y_{exp}$ are the gene expression values; $X_{ess}$ and $Y_{ess}$ are the gene essentiality values; and ⊕ represents the concatenation operation.

#### Network encoder.

To enhance feature generation for individual genes, we utilized features derived from a biological graph to represent genes as indicators. This is crucial because relying solely on multi-omics values may obscure the necessary semantic meaning. To incorporate this biological context with gene features effectively, we introduced a network encoder that leverages the GraphSAGE algorithm [29] to integrate the physical protein-protein interaction network. The algorithm is powerful for learning node representations in large graphs, adeptly capturing the local graph structure by sampling a fixed-size neighborhood around each node and then aggregating the sampled information from neighboring nodes using either mean or long short-term memory (LSTM) aggregation functions. For the input network, we employed a two-layer SAGE-convolutional network with 256 and 16 hidden layers to model gene relations defined by the local topology and generate gene representations. Here, we utilized the mean aggregation function. As part of the graph input, we utilized gene profiles across all cell lines from the CCLE dataset and applied principal component analysis. Specifically, PCA was performed on the all genes × all cell_lines matrix to extract the top 128 principal components for both gene expression and gene essentiality. These were then concatenated to form a final 256-dimensional feature vector for each gene, and it is consistent for all genes and cell lines:


$$F_{g}=\sigma (W_{\text{1}}F_{G}+W_{\text{2}}\cdot {mean}_{j\in ℵ(i)}F_{j})$$ (2)

where $W_{\text{1}}$ and $W_{\text{2}}$ are the learned parameters of the graph convolution network; $F_{G}$ is the input node feature; $F_{j}$ is the node feature of sampled local neighbors; and σ is the activation function, e.g., LeakyReLU.

#### Cell-line encoder.

To investigate the predictive capability of the proposed method for estimating GCEs in novel cell lines, we included the cell-line encoder as an indicator. By learning the cell-line features, the encoder enhanced the prediction of the final gene combination score. As its input, we utilized multi-omics values, such as gene essentiality and gene expression, across all genes. Subsequently, we applied PCA to reduce the dimensionality to 128 dimensions. Specifically, PCA was performed on the all cell_lines×all genes matrix to extract the top 128 principal components for both gene expression and gene essentiality. Here, we used two two-layer multi-layer perceptron (MLP) models to encode the cell-line feature:


$$F_{c\_ess}={MLP}_{ess}(C_{ess})$$ (3)

$$F_{c\_exp}={MLP}_{exp}(C_{exp})$$ (4)

where $C_{ess}$ and $C_{exp}$ are the corresponding cell-line representations.

#### Predictor and loss function.

Using the features generated from the omics, gene, and cell-line encoders, we utilized a multi-layer perceptron model to integrate combination of their outputs for predicting the final gene combination score. Because the score’s prediction involves a regression task, we employed the mean squared error (MSE) as the loss function for optimization:


$$z=MLP([F_{omics}⊕F_{g}⊕F_{c\_ess}⊕F_{c\_exp}])$$ (5)

$$z=MLP([F_{omics}⊕F_{g}])$$ (6)

$$z=MLP([F_{omics}⊕F_{c\_ess}⊕F_{c\_exp}])$$ (7)

$$\begin{array}{c} 𝓁=\Phi (z,y) \end{array}$$ (8)

where Φ is the MSE function, and y denotes the ground truth gene combination score.

### Model evaluation and implementation

To evaluate the effectiveness of our proposed method, we conducted experiments on CDKO benchmark. We begin by presenting our experimental setup, followed by the presentation of experimental results.

#### Setup of computation experiment.

The first computation experiment is leave-one-out to evaluate the generalizability of the proposed model for predicting novel GCEs in a new cell. It used data from 17 of 18 cell lines for training, and data from one left-out cell for external testing. During the model training, data from 16 cell lines were divided into internal training 95% and internal validation 5%. We further categorized the data into three distinct types for comprehensive evaluation, aiming to assess the method’s generalizability in predicting novel gene combination scores, as outlined below:

- C1: splitting by the gene combination pair, where the genes were shared between the training and testing sets.
- C2: splitting by the gene, where no same gene pairs were shared between training and testing sets, while one of the gene in a gene pair was shared between two data sets.
- C3: splitting by the gene, where none of the gene was shared between the training and testing sets.

#### Evaluation Metric.

We evaluated the model using the Pearson correlation coefficient, which quantifies the linear correlation between the predicted value and actual ground truth score:


$$r=\frac{\sum \left(x-\overset{―}{x})(y-\overset{―}{y}\right)}{\sqrt{\sum {\left(x-\overset{―}{x}\right)}^{\text{2}}{\left(y-\overset{―}{y}\right)}^{\text{2}}}}$$ (9)

where x is the value of the sample; $\overset{―}{x}$ is the mean of the samples; y is the ground truth value of the sample; and $\overset{―}{y}\;$ is the mean of the ground truth values.

### Implementation

Our data analysis investigated six different models: linear regression, Xgboost, multi-omics encoder, multi-omics encoder + network encoder, multi-omics encoder + cell line encoder, and the three encoders combined. We implemented the proposed method using scikit-learn, Pytorch, and PyG [30] packages in Python optimized using the Adam optimizer [31]. To address overfitting, we applied an early stopping technique in which training was ended if performance did not improve over 10 epochs. All experiments were conducted on an online cluster equipped with 88-core CPUs and NVIDIA Tesla A100 GPU with 40 GB RAM. The learning rate for all experiments was set at 0.001, 0.0025, 0.0025 and 0.0025, respectively. Each experiment was trained for 80 epochs in total. We employed a step-decay learning rate (StepLR) approach to adjust the learning rate, reducing it by gamma of 0.1 at the 50th epoch.

## Results

### Multi-omics feature selection in predicting gene combination effect

**Fig 4:**
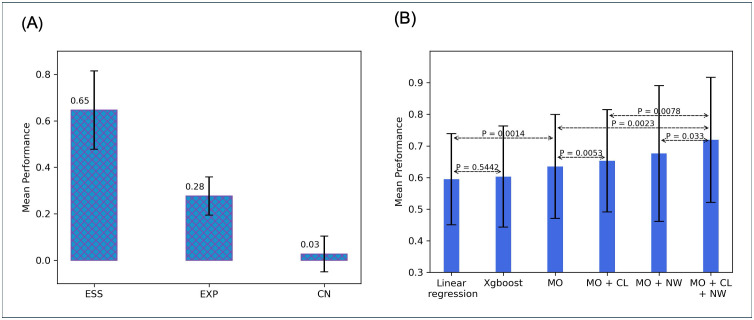
(A) shows results of omics feature selection for multi-omics encoder. (B) shows average prediction performance of different submodules in 18 cell lines.

For the key features for the gene combination score prediction, we compared gene expression, essentiality, and copy number as features for predicting the gene combination score using MLP, which is the same structure as multi-omics encoder. As shown Fig 4A, the y-axis represents the mean correlation between the predicted scores and the ground truth across 18 cell lines. Gene essentiality demonstrated the strongest predictive power for GCEs, followed by gene expression. In contrast, copy number showed limited predictive ability. Therefore, we selected gene expression and essentiality as the key inputs for multi-omics encoder. It also verify our hypothesis based on the Figs 2 and 3, where shows the sum of gene pair essentiality and expression have the strong correlation with gene combination score.

### Gene combination effect prediction performance for each cell

Table 2 summarizes the GCE prediction performance of our proposed methods for each individual cell. In this table, the GCE in one cell line is predicted from models that were trained on the other cell lines. The prediction performance is estimated as the correlation between the predicted GCE score and GCE score calculated from observed data in CDKO experiment. We investigated six models, including the linear regression, Xgboost, multi-omics encoder, multi-omics encoder plus network encoder, multi-omics encoder plus cell-line encoder, and all three encoders.

**Table 2:** Prediction performance of our proposed multi-layer encoder model to predict gene combination effect for each cell-line.

Cell-line	LR	XGBoost	MO	MO + CL	MO + NW	MO + CL + NW
22RV1	**0.750**	0.664	0.721	0.739	0.512	0.540
SAOS-2	0.573	0.591	**0.600**	0.576	0.434	0.458
JURKAT	0.194	0.207	0.211	0.213	**0.342**	0.331
K562	0.298	0.245	0.283	**0.325**	0.296	0.301
OVCAR8	0.574	0.508	0.561	0.583	0.362	**0.690**
A549	0.520	0.462	0.517	0.548	0.515	**0.603**
MELJUSO	0.608	0.641	0.678	0.703	0.802	**0.841**
HT29	0.634	0.757	0.794	0.745	**0.893**	0.861
A375	0.526	0.524	0.540	0.572	0.526	**0.610**
786O	0.780	0.748	0.814	0.813	**0.935**	0.887
HS944T	0.644	0.680	0.721	0.746	0.828	**0.878**
HS936T	0.659	0.682	0.720	0.762	0.841	**0.884**
HSC5	0.651	0.698	0.724	0.755	0.838	**0.880**
IPC298	0.704	0.744	0.777	0.804	0.842	**0.880**
MEL202	0.648	0.640	0.667	0.688	0.790	**0.830**
PATU8988S	0.651	0.662	0.696	0.728	0.825	**0.834**
PK1	0.632	0.671	0.675	0.715	0.792	**0.824**
GI1	0.664	0.682	0.738	0.744	0.793	**0.811**

LR (linear regression), MO (multi-omics encoder), CL (cell-line encoder), NW (network encoder). Bold values highlight the best performance among the submodules.

Notably, the strong linear relationship we observed between gene essentiality and expression with the gene combination score (Figs 2 and 3), especially in the 22Rv1 cell line, underscored the superior performance of linear regression over the other methods on 22Rv1. It was evident that linear regression effectively captured the linear relationship between gene expression/essentiality and gene combination score. In addition, the strong linear relationship between gene essentiality, gene expression, and the gene-combination score shows multi-omics encoder’s superior performance on SAOS-2 compared with other modules. In contrast, neither expression nor essentiality in JURKAT and K562 cells showed much correlation with the gene combination score. Therefore, all the models perform poorly in these two cells. In 12 of 18 cells, the optimal performance of the combined multi-omics encoder, cell line encoder, and network encoder model, i.e., correlation between predicted and observed GCEs, exceeded 80%, a result strongly supporting our hypothesis that deep learning methods have done an excellent job in predicting gene combination effect in about two-thirds of cells in our dataset. Overall, the combination of three encoders in MLEC-iGeneCombo show an average 71.9% prediction performance for a new cell.

### The cell-line and network encoders improved performance

Fig 4B shows the average prediction performance of the six models on gene combination score among the 18 cell lines. XGBoost shows only a minor improvement over linear regression (p = 0.5442) and even performs worse in some cases, suggesting that using multi-omics features alone is insufficient to capture the relationship between these features and gene combination scores. The superior prediction performance of the multi-omics encoder (63.5%) to that of linear regression (59.5%) (p = 0.0014) suggests that nonlinear relationships in the multi-omics encoder predict better than the linear regression model. This p-value was calculated from a paired t-test. Addition of the cell-line encoder to the multi-omics encoder increased prediction performance from 63.5% to 65.3% (p = 0.0053), and adding the network encoder to the multi-omics encoder increased the prediction performance from 63.5% to 67.6% (p = 0.1583). The 71.9% prediction performance of the three encoders together were significantly higher than that of the multi-omics encoder alone (p = 0.0023), also higher than only using two of three encoders (p = 0.0078 and p = 0.033). This evidence strongly supports the hypothesis that both the cell-line and network encoders contain information in predicting gene combination score.

### Performance in predicting gene combination effect depends on overlap of gene pairs between the training and testing Datasets

**Fig 5:**
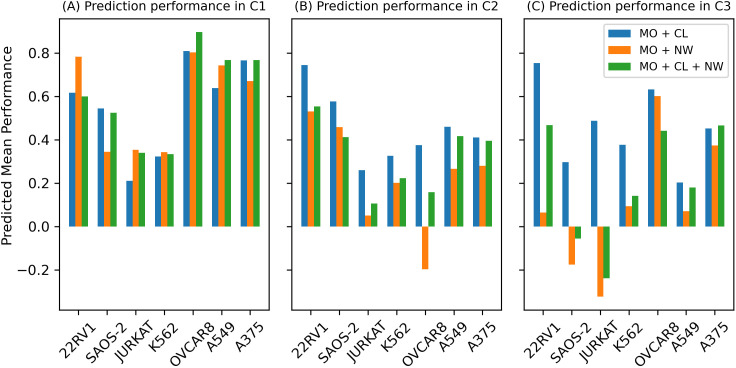
Testing results of separated sets C1, C2 and C3.

We further evaluated performance in predicting gene combination score by dividing testing data into three categories based on the overlap of genes and gene combinations. In the first group, C1, both paired genes overlapped between training and testing data; in the second, C2, only one gene in a pair overlapped between training and testing data; and in the third, C3, no gene overlap between training and testing data. Thus, only seven cell lines, as the testing set respectively, satisfy the splitting rule, while other cell lines as the testing set have a total overlap with the training set. We investigated the prediction performance of three models, those consisting of a multi-omics encoder + cell line encoder, a multi-omics encoder + network encoder, and all three encoders. Fig 5 shows that, in these seven cell-lines, only the multi-omics plus cell-line encoder shows comparable performance across the C1, C2, and C3 subsets.

- It is not surprised that predictions in C1 are uniformly better than C2 and C3, as training and validation sets have totally overlapped genes in C1, which C2 and C3 have only partial or no-overlapping genes.
- When training and validation sets have overlapping genes in C1, models with all three encoders, multi-omics, cell line and network, have the best performance.
- When training and validation sets have partial or non-overlapping genes in C2 and C3, models with only omics and cell encoders have better prediction performance than models with network encoder. In other words, network encoder makes it worse in predicting gene combination effect for new genes. This performance decline may be attributed to the network encoder based on graph topology, which may struggle to extract meaningful features for unseen gene nodes. Specifically, the network encoder relies on the protein-protein interaction (PPI) graph topology, and each node is represented by population-level gene features across all cell lines. During training, the model only observes part of the graph. As a result, it is difficult for the encoder to fully capture the global structure and generalize to unseen regions of the graph, especially when new genes are connected to subgraphs or neighbors that were never encountered during training. However, for cell lines containing genes that were present in the training set, the results remain strong. As shown in Table 2, the module of multi-omics encoder + network encoder shows 3 cell lines achieve the best performance across all models, and 8 cell lines perform better than all models except the one using all three encoders. And in Fig 4A, the mean performance of the multi-omics encoder combined with the network encoder is second only to the model using all three encoders, outperforming the other models. In S1 Appendix, we used Node2Vec to generate embeddings for the PPI network nodes and added these as additional features to the node inputs. The results show that this approach benefits cell lines containing novel genes do not present in the training set, suggesting that incorporating graph topology features aid in learning representations for new genes to some extent. For more details, please refer to S1 Appendix.

### Ablation Studies

**Fig 6:**
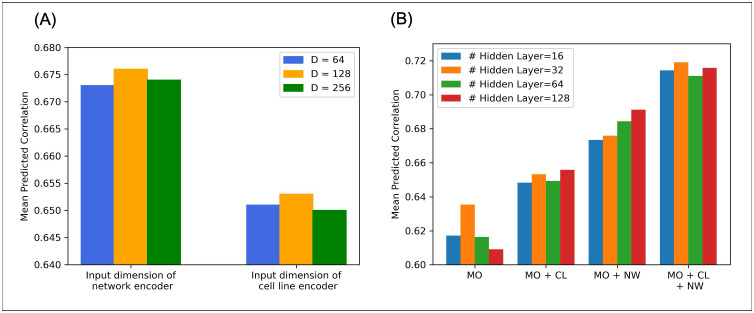
This figure includes results of all ablation studies. (A) shows results of input-layer ablation studies; (B) shows the results of hidden-layer ablation studies.

We performed ablation studies to select the optimal input dimensions of the network and cell-line encoders. Fig 6A shows the results of our evaluation of three choices of dimension, 64, 128, and 256 for module multi-omics encoder + cell-line encoder and module multi-omics encoder + network encoder, clearly showing that the use of 128 dimensions led to the highest average prediction performance across 18 cell lines in both the network and cell-line encoders. Therefore, in subsequent model development, input will consist of 128 dimensions of gene profiles for the network encoder and 128 dimensions of cell-line profiles for the cell-line encoder. We then performed additional ablation studies to select the dimension of hidden layers, studying 16, 32, 64, and 128 dimensions. We also studied four prediction models: the multi-omics encoder alone, the multi-omics encoder plus the cell-line encoder, the multi-omics encoder plus the network encoder, and the three encoders combined. Fig 6B clearly shows that 32 hidden layers showed the highest prediction performance among the three models and four hidden layer dimensions, except for multi-omics encoder + network encoder. Therefore, in subsequent experiments, we will choose a 32 hidden-layer configuration.

## Discussion

We developed the MLE-GeneCombo model, which includes multi-omics, network, and cell-line encoders, to predict GCEs in new cells, and we investigated the model’s prediction performance and that of its many sub-models using data of 18 cell lines from CDKO experiments. We showed that our MLE-GeneCombo model performed well in predicting GCEs, with prediction correlation above 80% in eleven of 18 cancer cells. It has an average GCE prediction performance of 71.9% for a new cell. MLEC-iGeneCombo fundamentally differs from existing SL prediction models in multiple aspects. First and far most, MLEC-iGeneCombo is the first model designed for predicting LFC, a new measurement of GCE. Secondly, MLEC-iGeneCombo can predict GCE in a new cell, while existing prediction models can only perform population based prediction [20–23,32–34] or predict gene combination effects for only one cell [24,25].

In studying the contributions of the three individual encoders in our MLEC-iGeneCombo model, we showed that the multi-omics encoder performed better than linear regression, and both the network and cell-line encoders were informative in the prediction of gene combination effect and enhanced the predictive performance of the multi-omics encoder alone. However, we recognized that the network encoder’s prediction of gene combination effect on new genes and new gene pairs could be misleading. It was likely due to the encoder’s inability to extrapolate GCE prediction from current gene sets and their representative network topology to new gene set, or network topologies between new genes and current genes are highly different. This remains a challenge and an opportunity for future research in GCE prediction.

The MLEC-iGeneCombo model also performed poorly in two cancer cells, K652 and JURKAT. This may be the result of the selection of only non-essential genes in the CDKO experiment between these two cells, which is not the case in other cell-line data. Perhaps the gene essentiality score will not predict well on the gene combination effect if both genes are non-essential. This can be an interesting hypothesis for future work.

## Supporting information

S1 Table Data Processing and Analysis of Published Synthetic Lethality Experiments. This table shows the data processing and analysis methods for synthetic lethality scores in published gene combination double knockout experiments.
(DOCX)

S2 Table 18 cell-lines information. This table shows the basic information for 18 cell-lines.
(DOCX)

S1 Appendix Supplementary materials. It provides additional details on how the gene. combination effect score is calculated and offer detailed descriptions of the gene combination double knockout experiments. Also, the node2Vec feature generation and results of adding the node2Vec feature to network encoder.
(DOCX)
