# Supplementary material for: A multi-layer encoder prediction model for individual sample specific gene combination effect (MLEC-iGeneCombo)
Source: PLoS Comput Biol. 2025 Oct 3;21(10):e1013547. doi: 10.1371/journal.pcbi.1013547 (PMC12510635; doi:10.1371/journal.pcbi.1013547)
Supplement: S1 Table — This table shows the data processing and analysis methods for synthetic lethality scores in published gene combination double knockout experiments. (DOCX) [file pcbi.1013547.s001.docx]

| **Study** | **Dual-gRNA count filtering** | **Dual-gRNA count normalization** | **Dual-gRNA effect** | **Dual-gRNA effect normalization** | **SL score calculation methods** |
| --- | --- | --- | --- | --- | --- |
| *Wong et al. 2016 [6]* | Dual-gRNA with less than 100 counts in day T were filtered out. | Normalized to total reads on sgRNA level | LFC of dual-gRNA counts between day 0 and day T. | NA | Mean LFC of dual-gRNA from a gene pair were compared to dual-gRNA with one gRNA target a gene and one control gRNA, in t-test. Average LFC of dual-gRNA for two genes < 0.9. |
| *Han et al. 2017 [7]* | Dual-gRNA with less than 50 counts were filtered out at day 0. At day T, Dual-gRNA with less than 50 counts were replaced with 1. | NA | LFC of dual-gRNA counts between day 0 and day T. | LFC is normalized to mean of LFC in controls. | Average LFC of dual-gRNA from a gene pair were compared to average LFC of dual-gRNAs, which has at least one control gRNA. It was analyzed in Mann Whitney U test. |
| *Shen et al. 2017 [9]* | NA | Normalized to cell population at day 0 and day end. | LFC of dual-gRNA counts between day 0 and day T. | LFC is normalized to mean of LFC in controls. | Average LFC of dual-gRNA from a gene pair were compared to the additive effect from two individual genes, in which the individual gene effect is averaged from dual-gRNA that has one control gRNA and one targeted gRNA. The statistical analysis was two-way ANOVA with interaction. The significance of statistical interaction was calculated as Bayes posterior probability. |
| *Horlbeck et al. 2018 [8]* | Pseudo-count of 10 for all dual-gRNA counts below 10. | Normalized to cell population at day 0 and day end. | LFC of dual-gRNA counts between day 0 and day T. | LFC is normalized to mean of LFC in controls. | Genetic interaction score was averaged among observed LFC of dual-gRNAs minus expected LFC of dual-gRNA, in which the dual-gRNA for two genes was fitted into quadratic linear regression of dual-gRNAs for single genes. |
| *Zhao et al. 2018 [10]* | NA | Normalized to cell population at day 0 and day end. | LFC of dual-gRNA counts between day 0 and day T. | LFC is normalized to mean of LFC in controls. | Average LFC of dual-gRNA from a gene pair were compared to the additive effect from two individual genes, in which the individual gene effect is averaged from dual-gRNA that has one control gRNA and one targeted gRNA. The statistical analysis was two-way ANOVA with interaction. The significance of statistical interaction was calculated as Bayes posterior probability. |
| *Ito et al. 2021 [11]* | Add a constant, 32, into the dual-gRNA reads. | Normalized to the total reads on dual-gRNA level. | LFC of dual-gRNA counts between day 0 and day T. | LFC is normalized to median of total LFCs. | SL scores were calculated from a hierarchical Bayesian model, **GEMINI**. In **GEMINI,** both additive effect from individual gene and genetic interaction effect between two genes were modeled. SL scores include strong lethality score and sensitive lethality and their FDRs. |
| *Parrish et al. 2021 [3]* | Dual-gRNAs less than 2 per million reads were removed. | Median ratio normalization | **MAGeCK**-calculated LFC. | LFC was normalized by setting the median of dual control sgRNA LFC to 0. | Average LFC of dual-gRNA from a gene pair were regressed on the average LFC of dual-gRNAs, which has at least one control gRNA. Benjamini-Hochberg FDR was calculated. |
| *Diehl, et al. 2021 [14]* | Dual-gRNAs with 0 read were removed. | Median ratio normalization | **MAGeCK**-calculated LFC. | LFC was normalized by setting the median of dual control sgRNA LFC to 0. | For each gene pair, five SL scores were calculated based on dual-gRNA LFCs: SUM, MIN, LOG, MULT, and MAX. |
| *Thompson et al. 2021 [12]* | NA | NA | LFC of dual-gRNA counts between day 0 and day T. | NA | LFC of dual-gRNAs from a gene pair were regressed on the additive LFC of dual-gRNAs from two individual genes. SL gene pairs were tested and ranked by t-test and robust rank algorithm. |
| *Tang et al. 2022 [5]* | Dual-gRNAs with 0 read were removed. | Normalized to the total reads on dual-gRNA level. | LFC of dual-gRNA counts between day 0 and day T. | NA | Average LFC of dual-gRNA from a gene pair were compared to the additive effect from two individual genes, in which the individual gene effect is averaged from dual-gRNA that has one control gRNA and one targeted gRNA. The statistical analysis was two-way ANOVA with interaction. |

Table 1. Data processing and analysis methods for synthetic lethality scores in published gene combination double knockout experiments
