# Supplementary material for: A multi-layer encoder prediction model for individual sample specific gene combination effect (MLEC-iGeneCombo)
Source: PLoS Comput Biol. 2025 Oct 3;21(10):e1013547. doi: 10.1371/journal.pcbi.1013547 (PMC12510635; doi:10.1371/journal.pcbi.1013547)
Supplement: S2 Table — This table shows the basic information for 18 cell-lines. (DOCX) [file pcbi.1013547.s002.docx]

Input Cell-lines. S2 Table summarizes the information of 18 cell lines.

S2 Table: Information of 18 cell lines.

| Cell-line | Cancer Type | Gene Pair Counts | Gene Counts | Correlation between Gene pair expression and gene combination score | Correlation between Gene pair essentiality and gene combination score |
| --- | --- | --- | --- | --- | --- |
| 22RV1 | Prostate | 1081 | 47 | -0.312 | 0.780 |
| JURKAT | Haematopoietic and lymphoid tissue | 58993 | 344 | -0.149 | 0.179 |
| K562 | Pleural effusion | 78210 | 396 | -0.180 | 0.284 |
| OVCAR8 | Ovary | 1334 | 70 | -0.116 | 0.653 |
| A549 | Lung | 8278 | 3004 | -0.304 | 0.530 |
| MELJUSO | Skin | 4617 | 2955 | -0.307 | 0.675 |
| HT29 | Large intestine | 253 | 23 | -0.293 | 0.598 |
| A375 | Skin | 1334 | 1504 | -0.236 | 0.561 |
| 786O | Kidney | 253 | 23 | -0.163 | 0.796 |
| HS944T | Skin | 4364 | 2949 | -0.303 | 0.737 |
| HS936T | Skin | 4364 | 2949 | -0.336 | 0.718 |
| HSC5 | Skin | 4364 | 2949 | -0.304 | 0.738 |
| IPC298 | Skin | 4364 | 2949 | -0.346 | 0.797 |
| MEL202 | Eye | 4364 | 2949 | -0.406 | 0.674 |
| PATU8988S | pancreatic adenocarcinoma | 4364 | 2949 | -0.338 | 0.705 |
| PK1 | Liver | 4364 | 2949 | -0.330 | 0.695 |
| GI1 | Gliosarcoma | 4364 | 2949 | -0.332 | 0.748 |
| SAOS-2 | Osteosarcoma | 1540 | 56 | -0.208 | 0.597 |
