# Supplementary material for: A multi-layer encoder prediction model for individual sample specific gene combination effect (MLEC-iGeneCombo)
Source: PLoS Comput Biol. 2025 Oct 3;21(10):e1013547. doi: 10.1371/journal.pcbi.1013547 (PMC12510635; doi:10.1371/journal.pcbi.1013547)
Supplement: S1 Appendix — It provides additional details on how the gene. combination effect score is calculated and offer detailed descriptions of the gene combination double knockout experiments. Also, the node2Vec feature generation and results of adding the node2Vec feature to network encoder. (DOCX) [file pcbi.1013547.s003.docx]

**Supplementary materials**

Here, we provide additional details on how the gene combination effect score is calculated, which were omitted from the main text and included in this appendix. We also offer detailed descriptions of the gene combination double knockout experiments. And for explore why the network encoder cannot performance well for new genes, we include results from incorporating node2vec-derived graph features as additional node inputs.

**
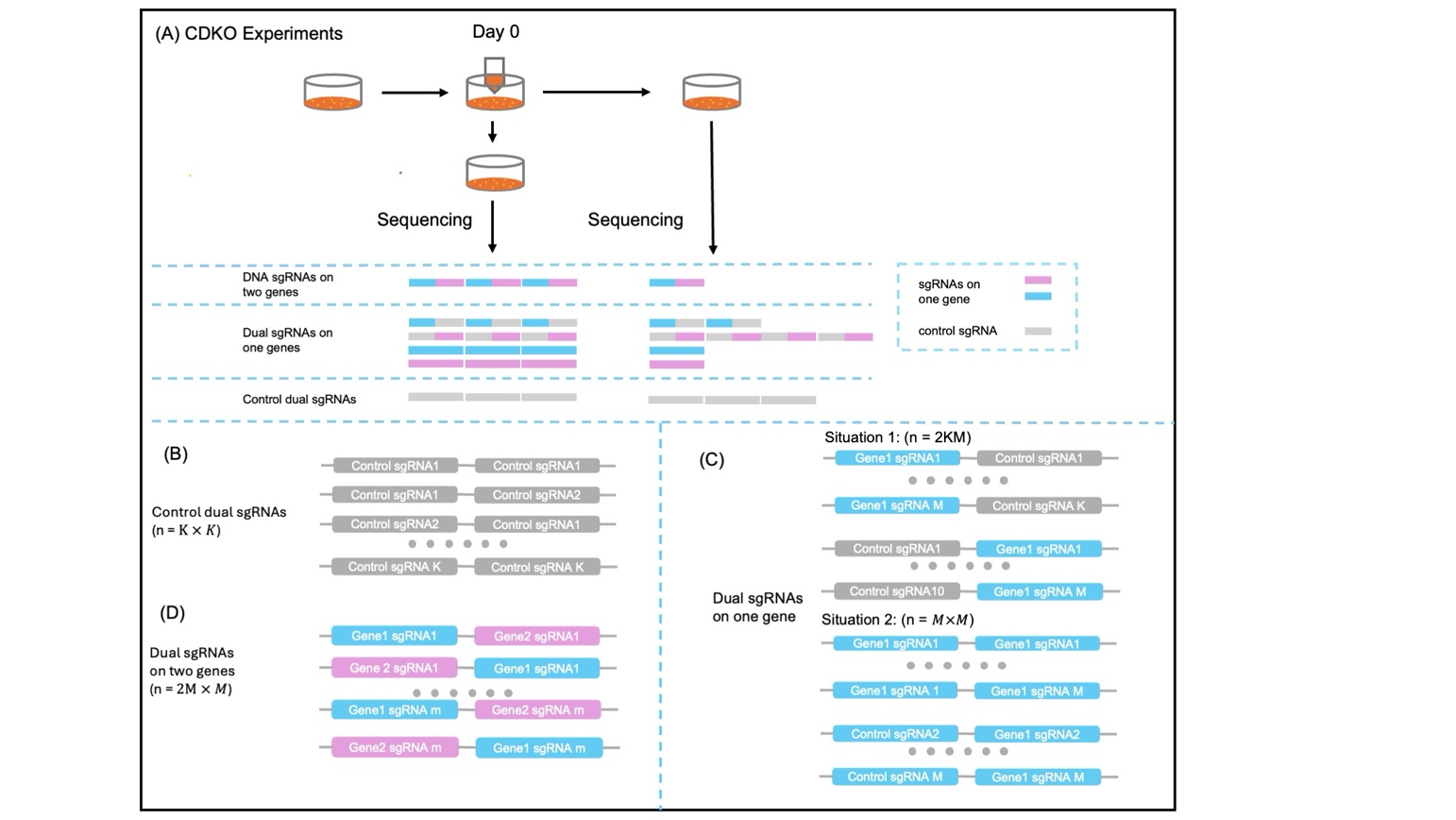
CDKO Experiments**. Cancer cells are infected with a lentivirus that contains a library of dual sgRNAs at Day 0 Figure 1A, at which time the sgRNA sequences are approximately evenly distributed. After a period of usually three to four cell cycles, at Day $T_{End}$, significant suppression of cell viability by the dual-sgRNA-guided CRISPR-cas9 gene knockout will be reflected in a decrease in the corresponding dual-sgRNA sequences from Day 0. Thus, the fold change in the dual-sgRNA sequences from Day 0 to Day $T_{End}$ indicates the effect of the dual sgRNAs on cell viability.

Figure 1: Synthetic lethality experiment and dual-sgRAN data for target on a control gene, one gene, and two genes

Without loss of generality, let us assume there are $K$control sgRNAs, and $M$ sgRNAs are targeting a gene in the design. Figure 1B illustrates that there are $K^{2}$ control dual sgRNAs. Figure 1C shows two types of dual sgRNAs targeting one gene. In the first scenario, one control sgRNA and one sgRNA target a gene ($2MK)$, and in the second scenario, two sgRNAs target the same gene $(M^{2})$. Figure 1D shows dual sgRNAs targeting two genes. If we consider orientations, $2M^{2}$ dual sgRNAs are targeting one gene pair.

Let $g\in\left\{ 1, 2, .. , G \right\}$ denote an sgRNA target gene, where $G$ is the total number of unique gene targets. Let $t \in\{T_{0}, T_{end}\}$ describe the experiment starting time point $\left( T_{0} \right)$ and experiment ending time point $\left( T_{End} \right)$. Let $i \in\{1, 2, .. , I\}$ denote the index of sample replicates. In CDKO experiments, a construct has a pair of sgRNAs. The sgRNA

count, $Y$, is differentially annotated according to the targeting of its sgRNA pair; two genes, one gene, or no gene, i.e., the control.

- Control sgRNA pair: If both sgRNAs target either a non-human gene or intergenic human genome, there are $S_{0}$ = $K^{2}$such sgRNA pairs, and the count of a control sgRNA pair is denoted as $Y_{0,sti}$, where $s=\{1,\ldots,S_{0}\}$. Usually, log fold changes (LFCs) of $Y_{0,sti}$s are analyzed directly, $X_{0,si}=log(\frac{Y_{0,sT_{end}i}}{Y_{0,sT_{0}i}})$.
- Single-gene sgRNA pair: If both sgRNAs target the same human gene or one sgRNA targets one gene and the other sgRNA targets a non-human gene or intergenic human genome, there are $S_{1}$ = $2MK$ + $M^{2}$such sgRNA pairs, and a single-gene sgRNA pair count is denoted as $Y_{1,gsti}$, where $s=\{1,\ldots,S_{1}\}$. The LFCs of $Y_{1,gsti}$ are $X_{1,gsi}=log(\frac{Y_{1,gsT_{end}i}}{Y_{1,gsT_{0}i}})$.
- Two-gene sgRNA pair: If two sgRNAs target two different human genes, there are $S_{2}$=$2M^{2}$ such sgRNA pairs, and a two-gene sgRNA pair count is denoted as $Y_{{2,g}_{1}g_{2}sti}$, where $s=\{1,\ldots,S_{2}\}$. The LFCs of $Y_{{2,g}_{1}g_{2}sti}$s are $X_{{2,g}_{1}g_{2}si}=log(\frac{Y_{{2,g}_{1}g_{2}sT_{end}i}}{Y_{{2,g}_{1}g_{2}sT_{0}i}})$

**Gene pair gene combination effect score =** median${\{X}_{{2,g}_{1}g_{2}si}=\log\left( \frac{Y_{{2,g}_{1}g_{2}sT_{end}i}}{Y_{{2,g}_{1}g_{2}sT_{0}i}} \right), s=\{1,\ldots,S_{2}\}\}$.

**Node2vec Feature Generation.** We represented the physical-interaction PPI network as an undirected graph and learned low-dimensional gene embeddings with Node2Vec. Node2Vec performs biased random walks and optimizes a skip-gram objective so that genes appearing in similar network contexts receive similar vectors. We set the embedding dimensionality to 128, random-walk length was 20 and the context window 10, which together capture both immediate neighbors and medium-range topological information. The return and in-out parameters were both fixed at p and q is 1. Embeddings were trained for 40 epochs with the SparseAdam optimizer and learning rate is 0.01.

**Results from Incorporating Node2Vec Features into the Network Encoder**. We appended the Node2Vec feature to the population-level gene expression and gene essentiality features, creating a 384-dimensional vector. Figure 2 shows the results of using the multi-omics encoder combined with the network encoder, with and without the Node2Vec feature, for six cell lines containing non-overlapping genes with the training set and eleven cell lines whose genes fully overlap with the training set.

The six cell lines show moderate improvement, while the other eleven exhibit similar results with or without the added Node2Vec features. This suggests that the approach is particularly beneficial for cell lines containing novel genes not seen during training, indicating that incorporating graph topology features can help in learning representations for new genes to some extent. Without the Node2Vec feature, the network encoder struggles to capture topological information effectively, resulting in poor generalization to unseen genes.


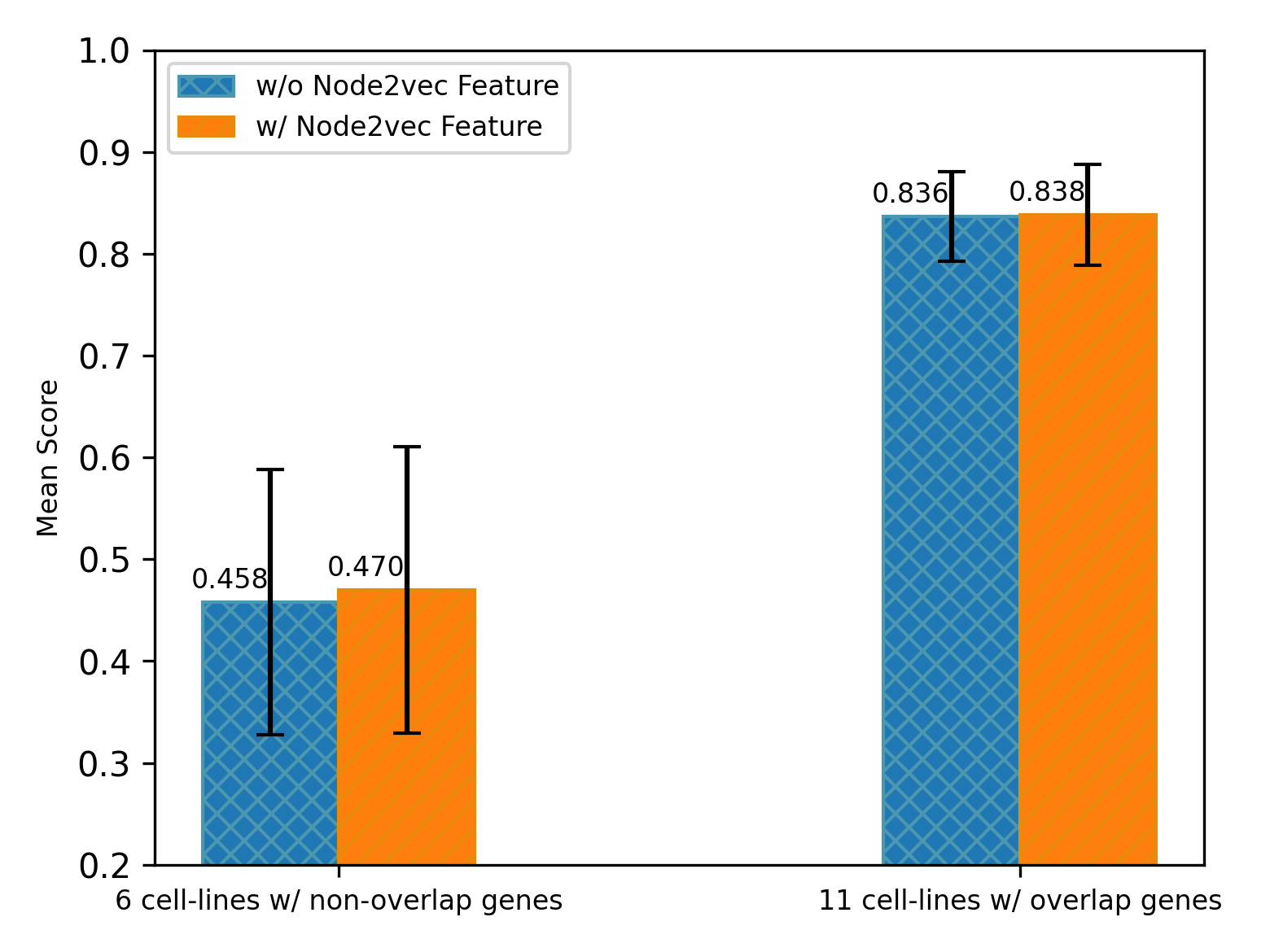


Figure 2: the results of using the multi-omics encoder combined with the network encoder, with and without the Node2Vec feature, for six cell lines containing non-overlapping genes with the training set and eleven cell lines whose genes fully overlap with the training set.
